# Encapsulated papillary carcinoma: Diagnosis, treatment, and prognostic outcomes: A report of 2 case report

**DOI:** 10.1097/MD.0000000000042339

**Published:** 2025-05-02

**Authors:** Mohammad Al-Jawad, Mohamed Nizar Akil, Mohammad Hassan Nabhan, Danah Abdulaziz, Leen Kanaa, Nazima Ali Bek, Ammar Niazi

**Affiliations:** a Faculty of Medicine, University of Aleppo, Aleppo, Syrian Arab Republic.

**Keywords:** case report, encapsulated papillary carcinoma, papillary carcinoma in situ, surgical excision

## Abstract

**Rationale:**

Encapsulated papillary carcinoma (EPC) is a rare subtype of breast cancer characterized by a favorable prognosis; however, its management can be complex, especially when associated with ductal carcinoma in situ (CIS) or invasive components. This study aimed to highlight the clinical presentation, diagnostic approach, and treatment outcomes of EPC to improve understanding and management strategies for this unique breast cancer subtype.

**Patient concerns:**

We present 2 cases of elderly women who presented with breast lumps, prompting concerns regarding the nature of the lesions and the need for effective management strategies.

**Diagnoses:**

Case 1 involved a 67-year-old woman diagnosed with EPC within an inflammatory cyst wall, while case 2 was an 85-year-old woman with EPC and low-grade papillary CIS. Both diagnoses were confirmed through imaging and histopathological examination.

**Intervention:**

Both patients underwent complete surgical resection of the tumors. Case 1 required no additional therapy postsurgery, while case 2 was monitored regularly without further treatment.

**Outcome:**

Both patients remained healthy following their respective interventions, with no evidence of recurrence noted during follow-up.

**Lesson:**

Early diagnosis and appropriate surgical intervention are critical in managing EPC. Regular monitoring is essential, particularly for cases with associated ductal CIS or invasive components, to ensure favorable outcomes.

## 1. Introduction

The disease known as encapsulated papillary carcinoma (EPC) of the breast, formerly referred to as intracystic papillary carcinoma, has been incorporated into the latest World Health Organization classification.^[1]^

EPC is a rare form of breast cancer that typically has a good prognosis. It is most commonly identified through screening mammograms or may present as a palpable mass.^[2]^

On the basis of histological characteristics, EPC can be classified into three subtypes: pure EPC, EPC associated with ductal carcinoma in situ (DCIS), and EPC associated with invasive carcinoma. Pure EPC, which lacks adjacent DCIS or invasive components, has an excellent prognosis with appropriate local treatment. However, when DCIS or invasive components are present, the risk of local recurrence increases significantly.^[3]^

In our study, we present 2 cases of EPC in female patients, both of which were successfully treated. The first patient, a 67-year-old woman, was diagnosed with EPC within an inflammatory cyst wall and underwent complete surgical resection, requiring no additional therapy and showing no signs of recurrence during follow-up. The second patient, an 85-year-old woman, was diagnosed with EPC and low-grade papillary carcinoma in situ (CIS). She also received a complete surgical excision and was monitored regularly without further treatment, with no recurrence noted. These cases highlight successful surgical outcomes and a favorable prognosis, contributing to an improved quality of life for both patients, this work has been reported in line with the CARE criteria.^[4]^

## 2. Case presentation

### 2.1. Case 1

A 67-year-old female patient presented with a progressively enlarging lump in the right breast. Notably, she exhibited no fever, localized signs of infection, or systemic symptoms. Her medical history included hypertension, type II diabetes, and renal insufficiency, for which she was receiving antihypertensive medications, diabetes management drugs, and diuretics.

The patient had a history of 3 abscess drainages in the right breast, the most recent occurring 3 years before. On physical examination, a tender mass was palpated in the right lower outer quadrant of the breast. Vital signs revealed a blood pressure of 140/98 mm Hg, a heart rate of 105 beats per minute, a temperature of 37.9°C, and an oxygen saturation (SpO_2_) of 98% on room air.

Laboratory tests indicated a complete blood count (CBC) showing a decrease in hemoglobin to 11.0 g/dl (normal range: 11.5–15.5 g/dl) and a hematocrit of 34.8% (normal range: 36–48%). The white blood cell and platelet counts were within normal limits. Red blood cell indices were elevated, with a red blood cell distribution width—coefficient of variation of 14.8% (normal range: 11.6–14.4%) and a red blood cell distribution width—standard deviation of 46.4 fL (normal range: 35–46 fL). Other biochemical parameters showed hyperglycemia with a fasting glucose level of 199 mg/dL (normal range: 70–110 mg/dL), elevated urea at 92 mg/dL (normal range: 15–44 mg/dL), and elevated creatinine at 2.03 mg/dL (normal range for adults: 0.5–1 mg/dL).

**Figure 1.**
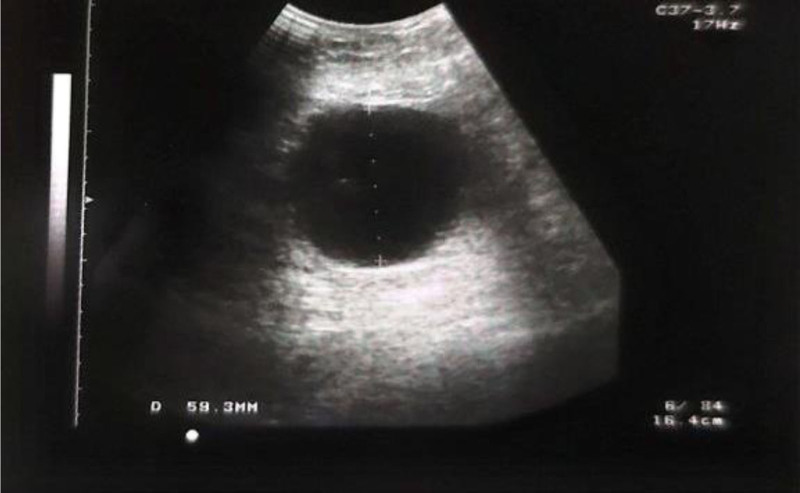
Ultrasound view of the complex mass/cyst.

**Figure 2.**
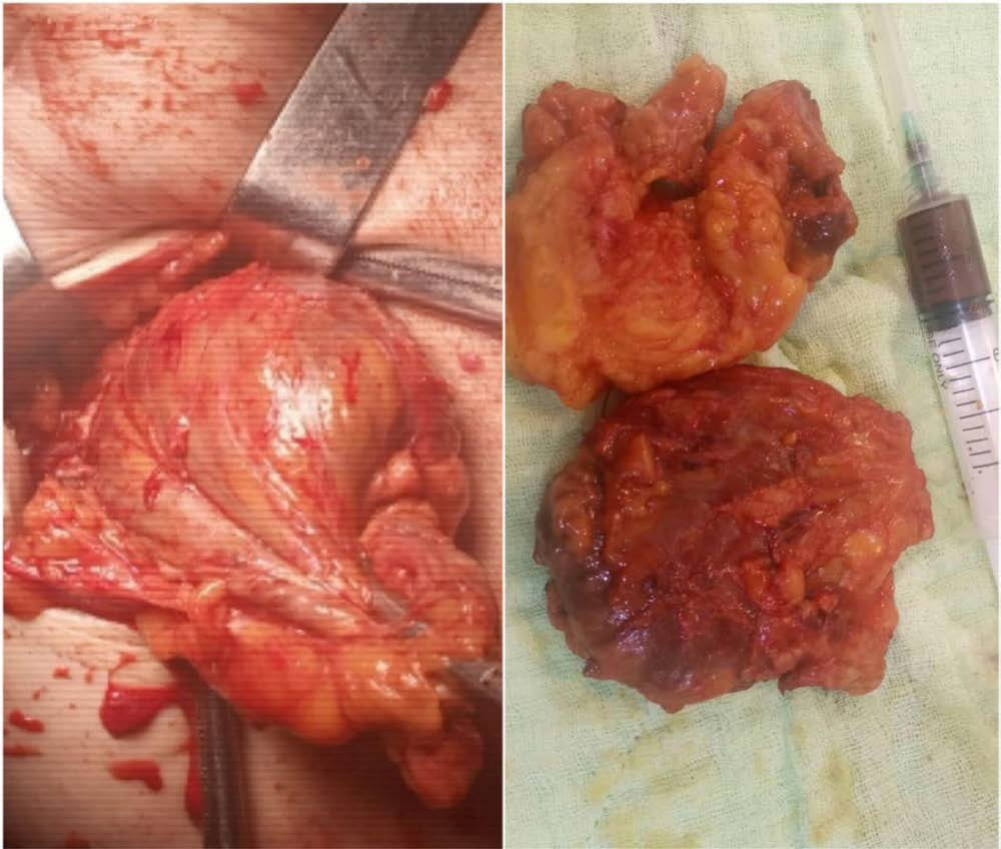
Image showing the surgical excision of a cyst containing encapsulated papillary carcinoma.

Ultrasonography revealed a 7 × 8 cm cystic lesion in the right lower outer quadrant of the breast containing turbid fluid Fig. 1. Mammography was not performed in this case. The patient subsequently underwent surgical excision of the cyst Fig. 2, and pathological examination revealed a hemorrhagic inflammatory cyst wall with EPC.

Postoperatively, the patient was in good health and did not require chemotherapy or radiotherapy following the surgical intervention.

## 3. Case 2

An 85-year-old woman presented to the hospital with a left breast lump that had developed over the past 6 months, gradually increasing in size. The lump was accompanied by normal nipple discharge, with no signs of fever, local inflammation, or enlarged lymph nodes in the breast tissue or left axilla. The right breast exhibited no abnormalities.

The patient’s medical history included type II diabetes and hypertension, along with a surgical history of cholecystectomy performed 14 years ago. On clinical examination, her heart rate was 76 beats per minute, and her blood pressure was recorded at 140/90 mm Hg.

**Figure 3.**
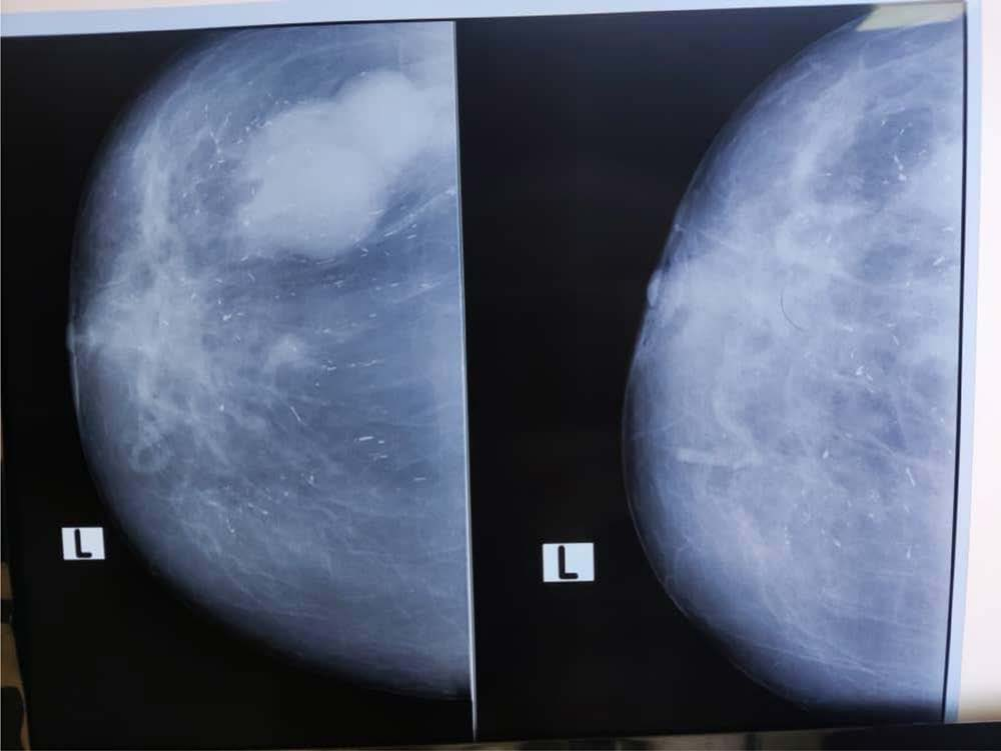
Image of mammography showing slightly heterogeneous breast tissue with lobular density at the 10–11 o’clock position and numerous small linear calcifications.

Mammography revealed slightly heterogeneous breast tissue, with a lobular density noted at the 10–11 o’clock position, accompanied by numerous small linear calcifications dispersed throughout the breast (Fig. 3). There was no evidence of skin thickening or nipple retraction.

Ultrasonography demonstrated a mixed mass lesion at the 10–11 o’clock position, consisting of a vascular tissue component and a cystic component, the largest measuring 2.3 × 2.6 cm. The overall size of the mixed lesion was 3 × 5 cm, exhibiting posterior acoustic enhancement and lobulated edges.

**Figure 4.**
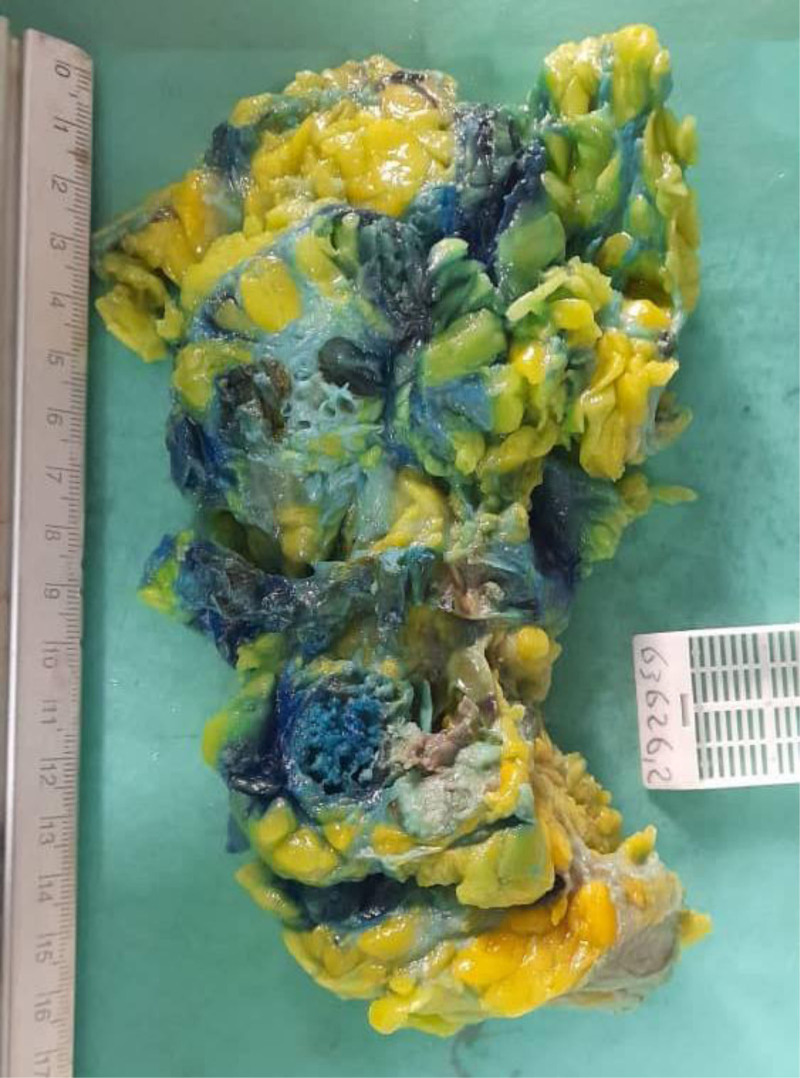
Image showing the gross examination postsurgical excision of the cyst, with the mass measuring 15 × 10 × 3 cm and a surgical cavity measuring 7 cm.

The patient subsequently underwent surgical excision of the cyst. On gross examination postexcision, the mass measured 15 × 10 × 3 cm, with the cut section revealing a surgical cavity measuring 7 cm (Fig. 4).

Microscopic examination of the tissue sections demonstrated cystic formation with papillary proliferation, characterized by ramified papillary structures lined by layers of epithelial cells exhibiting moderate eosinophilic cytoplasm and round nuclei. The predominant growth pattern was solid and sheet-like, with serial sections showing no evidence of an invasive carcinoma component. The adjacent parenchyma displayed dense interlobular, perilobular, and perivascular lymphocytic infiltrates, associated with lobular atrophy and areas of collagen deposition.

The final conclusion indicated that the mass is an EPC with a significant component of low-grade papillary CIS.

Postoperatively, the patient was in good health and did not require chemotherapy or radiotherapy following the surgical intervention. She has been monitored regularly for a full year, with ongoing assessments of her overall health and subsequent test results. Currently, her health remains excellent during follow-up, with no evidence of tumor recurrence.

## 4. Discussion

Papillary carcinoma of the breast is a rare subtype of invasive breast cancer, accounting for <1% of new diagnoses. It is characterized by its branching fibrovascular framework and is defined by well-defined lesions encased in a fibrous capsule, lacking myoepithelial cells at the periphery and within the papillae. Previous research has indicated that EPC predominantly affects elderly women, typically presenting as a subareolar mass and/or with nipple discharge.^[3,5]^

EPC typically presents as a large tumor, averaging around 2 cm, located within a sizable cystic duct. Clinically, EPC appears as a painless breast lump that may persist for several years. A common symptom is bloody nipple discharge, although it can often be asymptomatic and detected through screening mammography. Upon gross examination, it appears as a tan-white, well-defined, and friable tumor within a cyst.^[6]^

In case 1, a 67-year-old female presented with a progressively enlarging right breast lump, without fever or signs of infection. In case 2, an 85-year-old woman developed a left breast lump over 6 months, accompanied by normal nipple discharge and no signs of inflammation.

To diagnose this type of tumor, it typically presents as a solid mass or as a heterogeneous tumor containing both cystic and solid components. The margins are often well-defined, but sometimes the mass may have loosely bordered or multicircular contours, which could indicate malignancy. Internal echoes are caused by septations within the cystic part, and the presence or absence of vascularity is assessed using Doppler imaging.^[7–9]^

Regarding MRI, there are no specific features for EPC. However, it may present as an enhancing complex cyst or a multicystic lesion with a solid central component. MRI plays a crucial role in differentiating breast malignancies from benign lesions, especially when conventional imaging modalities are challenging.^[7–9]^

The diagnosis in both of our cases was made through a combination of imaging studies and histopathological examination. In case 1, ultrasonography identified a cystic lesion, which was followed by surgical excision, revealing EPC in the inflammatory cyst wall. In case 2, mammography and ultrasonography showed a mixed mass, and subsequent surgical excision confirmed EPC with low-grade papillary CIS. Both cases emphasized the critical role of imaging and pathology in accurate diagnosis.

We would like to clarify that while we have provided a comprehensive explanation of the pathological findings in our report, the pathology lab was unable to supply us with images due to the instability in our country. Furthermore, in case 1, the patient had previously undergone 3 abscess drainage procedures in the breast, resulting in calcifications that are difficult to detect on standard mammography. Unfortunately, high-definition mammography, which can identify such issues, is not available in our country; therefore, it was not performed.

In addition, genetic or molecular testing for the tumor is currently unavailable due to the ongoing civil war. Even if these tests were accessible, the financial situation of most residents makes it challenging to afford such procedures. However, we assure you that we have included all relevant details from the pathology report to ensure an accurate and thorough understanding of the case.

Therapeutic recommendations for EPC remain controversial. When invasion is absent, EPC is evaluated and managed as an in situ disease, while invasive cases are classified and treated based on their invasive characteristics. The standard treatment is complete surgical resection, with sentinel lymph node biopsy recommended due to the low likelihood of lymph node metastasis. All forms of EPC, including pure cases, those associated with DCIS, and invasive cases, carry a risk of recurrence. Depending on specific criteria, hormonal therapy, chemotherapy, and radiotherapy may be considered, with adjuvant radiotherapy being an option when DCIS or invasion is present. In cases of histologically aggressive invasive tumors, adjuvant chemotherapy is the preferred treatment.^[10–13]^

In both of our cases, the primary treatment involved surgical excision of the breast lumps. For case 1, the patient underwent a lumpectomy, and postoperative pathology confirmed EPC. Given the nature of the tumor and the absence of aggressive features, no further treatment such as chemotherapy or radiotherapy was required.

In case 2, the patient also had a lumpectomy, which revealed EPC with low-grade papillary CIS. Similar to case 1, the tumor’s characteristics indicated that adjuvant therapies were unnecessary, and the patient was monitored for any signs of recurrence. Both cases highlight the effectiveness of surgical intervention in managing EPC without the need for additional treatments.

Our study has several limitations. Due to ongoing civil unrest, access to advanced imaging and genetic testing was restricted, potentially affecting diagnostic accuracy. The lack of high-definition mammography hindered lesion detection in case 1. Financial constraints may also prevent patients from seeking comprehensive care. Finally, the absence of detailed pathological images limits the visual representation of findings. These factors should be considered when interpreting our results.

## 5. Conclusion

EPC is a rare type of breast cancer characterized by a favorable prognosis, particularly when diagnosed early and treated appropriately. The presented cases emphasize the importance of imaging studies and histopathological examination in accurately diagnosing EPC. Surgical excision remains the primary treatment, with patients not requiring additional therapies due to the noninvasive nature of their tumors. Regular monitoring is essential to detect any potential recurrence, especially in cases associated with DCIS or invasive components. Overall, early detection and timely intervention are crucial for optimizing outcomes in patients with EPC.

## Author contributions

**Methodology:** Mohammad Al-Jawad.

**Writing – original draft:** Mohammad Al-Jawad, Danah Abdulaziz, Leen Kanaa MS, Nazima Ali Bek.

**Writing – review & editing:** Mohammad Al-Jawad, Mohammad Hassan Nabhan.

**Supervision:** Mohamed Nizar Akil, Ammar Niazi.
